# Editorials

**Published:** 1910-11

**Authors:** 


### EDITORIALS

The Business Office of the *JOURNAL-RECORD* is 1 1-2 to 5 1-2 South Broad Street.
The Editorial Office is 1014-15 Century Building.
Address all Business Communications to *Journal-Record of Medicine*, 1 1-2 to 5 1-2 South Broad Street.
Make remittances payable to *THE JOURNAL-RECORD OF MEDICINE*.
On matters pertaining to the Editorial and Original Communications, address Edgar G. Ballenger, M. D., Atlanta. Ga.
Reprints of Original Articles will be furnished at cost price. Requests for the same should always be made in THE MANUSCRIPT.
We will present, postpaid, on request, to each contributor of an original article, twenty (20) marked copies of *THE JOURNAL-RECORD OF MEDICINE* containing such article.

### AGAIN "606."

Reports come thick and fast concerning the value of "606," the Ehrlich-Hata preparation, in the treatment of Syphilis. This remedy undoubtedly is the center of medical thought to-day—and deservedly. After seeing many hundreds of cases, in German clinics, who had been and were being treated with "606," the writer has no hesitation in stating that it is the most remarkable remedy ever discovered by the medical profession. The spirochetes frequently disappear like magic after a single injection of this remedy, and as far as can be judged from 13,000 cases treated since last March, it is safe to say it has come to stay. Certainly no other plan of treatment has ever effected such remarkable cures of any disease as has "606" in spirochetal affections—especially in syphilis. Of course as might of necessity have been expected, it has its contra-indications and limitations, but even in this respect it may be said to exceed reasonable expectations. The remarkable part has been the almost uniform results reported by a very large number of independent investigators. They all record the prompt disappearance of spirochetes from nearly all lesions, and wonderfully rapid healing. The patients gain in weight and have a feeling of well-being that certainly speaks well for the remedy. A few cases have been reported where no beneficial effects were noted after by the injection of "606," or with mercury. Why this should be the case has not been ascertained, but, fortunately, such patients have been very scarce. About 12 fatalities in adults have been reported as following the administration of this remedy, but most of these could be attributed to the almost moribund condition of the patients at the time of the injection, or to serious organic affections, which rendered the patients unsuitable subjects for the treatment. When one sees some of the patients who receive the treatment, it seems more wonderful than ever that more fatalities have not followed its use.

In walking through the wards and observing the patients, the observer is at once struck with the fact that they look so well and happy, and that progressing syphilitic lesions are not to be seen. A few patients have sore backs from necrosis which sometimes follows the injections, but aside from the discomfort occasioned, it does not militate against a successful outcome of the treatment. Nearly all patients have, for a few weeks to a few months, an induration at the site of the injection, but, after the first week, this causes practically no pain. How well cured the patients, treated with this remedy, really are, can only be judged absolutely after many years. One thing is certain, and that is, more good can usually be done with one or two injections than could be obtained after long courses of mercury or potassium iodid.

The Wassermann reaction becomes negative in a good percentage of cases and has remained so to the present in many. The reports and observations vary considerably as to the time the reaction becomes negative; usually this occurs from the second to the fifth week. It becomes negative in from 30 to 60 per cent. of the cases after a single injection. Fortunately, when needed, the second and third doses of "606" seem better borne by the patient than the first injection. Malignant forms of syphilis respond especially well and to smaller doses that the initial lesion before the rash has broken out. This is probably due to the formation of antitoxins by the germs killed by the arsenobenzol. That antitoxins are formed is shown by the manner in which syphilitic infants quickly improve, when nursing the mother's milk after she has received an injection of "606." The milk contains a trace of arsenic—much too little to cause such a rapid clearing of symptoms. Many women have received the treatment in the last stages of pregnancy, without any tendency toward miscarriage, and with the normal delivery of healthy babies.

Much bitterness of feeling exists in Germany over this remedy—attributable, perhaps, to jealousy, or to injured pride, because none of it had been given to many prominent men to test. In France, too, there has been considerable unfavorable criticism; this had been led by Hollapeau, who has a method of his own, by which he claims to abort *beginning* syphilis in 30 days. Unfavorable reports have also originated in Aix-la-Chapelle, the "Hot Springs" of the continent, where there will result a great financial loss from quick cures of syphilis by "606." There is also some feeling against Ehrlich, because he is a Jew, and because he has intrusted much of the experimental work to Jews. Knowing these facts enables one to judge better the actual value of the criticisms of "606," which must of necessity have its limitations and contra-indications.

It is given in doses ranging from 0.4 to 0.7 gramme, according to the weight and condition of the patient. The method of preparing it varies greatly according to whether it is to be administered subcutaneously, intramuscularly or intravenously. Each method has its advantages and disadvantages. Wechselmann, who has treated more patients than any other physician prefers the subcutaneous injection of the neutral emulsion, in the back, just below the scapula. For further details the readers are referred to the books on the subject, and to the numerous articles that are now appearing and have appeared during the past few months in so many medical journals. E. G. B.

---

### ECZEMA SPECIALISTS AND THEIR METHODS.

The following letter sent us by Dr. G. Y. Pierce explains itself and shows to what extent quacks will go in procuring the names of prospective "lambs." Is it too much to hope that someday the postal department will forbid the use of the mails to such fakirs?

Sedalia, Mo., Oct. 9th., 1910.

Red Cross Pharmacy, or Dr. G. Y. Pierce,
Atlanta, Ga.

Dear Sir:—

I want to BUY,—not sell.

I have treated nothing but ECZEMA, for EIGHT years.

Those that I treat can deposit their money in the bank, subject to the results of my treatment.

You no doubt know the names and addresses of those having eczema, and if you will send them to me, I will pay you $2.00 for each name as they deposit their money in the bank. If I fail, you keep the $2.00; if I do NOT fail, you keep it just the same.

It would surprise you what I give away this way every day.

Please notice—You do not invest one penny, not even a stamp, for I have supplied that. It is all profit to YOU, and certainly that is fair.

Your name will never be mentioned, for it is all I can do to write to these people, without going to the bother of telling them a lot of private affairs.

Just think of the quantity of proprietory medicine you would have to sell to each case to make $2.00 and you would have to put some money besides, in the medicine.—This way not a cent spent—ALL profit.

Your money will be sent you the very day the money is deposited in the bank, if nothing else, I must keep my word with those who send me these names, I simply can't AFFORD to do only the right thing.

In case TWO send the same name, the one sending it FIRST gets the $2.00. That is why we keep the envelope that carried the name. This has happened THREE times in THIRTY THOUSAND cases.

Use the enclosed envelope, and send them, and make some easy money, or throw it all in the waste basket and LOSE money.

Won't you please attend to this NOW, don't lay it down, for you will forget it sure. "DO IT NOW." "DO IT NOW."

Yours truly,

J. E. Cannaday, M. D.

P. S. It would be a paying thing for you to keep the names of parties you hear of and send them from time to time. Certainly you can be well prepaid for your trouble, other are—why not you?

